# Occurrence and Distribution of Tomato Brown Rugose Fruit Virus Infecting Tomato Crop in Saudi Arabia

**DOI:** 10.3390/plants11223157

**Published:** 2022-11-18

**Authors:** Ahmed Sabra, Mahmoud Ahmed Amer, Khadim Hussain, Adel Zakri, Ibrahim Mohammed Al-Shahwan, Mohammed Ali Al-Saleh

**Affiliations:** 1 Plant Protection Department, College of Food and Agriculture Sciences, King Saud University, Riyadh 11451, Saudi Arabia; 2 Botany Department, Faculty of Agriculture, Fayoum University, Fayoum 63514, Egypt; 3 Virus and Phytoplasma Research Department, Plant Pathology Research Institute, Agricultural Research Center, Giza 12619, Egypt; 4 Department of Bioinformatics and Biotechnology, Government College University Faisalabad, Faisalabad 38000, Pakistan; 5 Department of Plant Production, College of Food and Agriculture Sciences, King Saud University, Riyadh 11451, Saudi Arabia

**Keywords:** tomato, ToBRFV, Tobamovirus, plant virus, serological diagnosis, molecular diagnosis, disease severity index

## Abstract

During the growing season of 2021–2022, a total of 145 symptomatic tomato leaf and fruit samples were collected from different locations in Riyadh Region, Saudi Arabia, showing a moderate-to-severe mosaic with dark green wrinkling, blistering, narrowing, and deformation with necrosis spot on tomato leaves, while irregular brown necrotic lesions, deformation, and yellowing spots rendering the fruits non-marketable were observed on tomato fruits. These samples were tested serologically against important tomato viruses using enzyme-linked immunosorbent assay (ELISA), and the obtained results showed that 52.4% of symptomatic tomato samples were found positive for Tomato brown rugose fruit virus (ToBRFV), wherein 12 out of 76 samples were singly infected; however, 64 out of 145 had mixed infection. A sample with a single infection of ToBRFV was used for mechanical inoculation into a range of different host plants; symptoms were observed weekly, and the presence of the ToBRFV was confirmed by ELISA and reverse transcription–polymerase chain reaction (RT-PCR). A total RNA was extracted from selected ELISA-positive samples, and RT-PCR was carried out using specific primers F-3666 and R-4718, which amplified a fragment of 1052 bp. RT-PCR products were sequenced in both directions, and partial genome nucleotide sequences were submitted to GenBank under the following accession numbers: MZ130501, MZ130502, and MZ130503. BLAST analysis of Saudi isolates of ToBRFV showed that the sequence shared nucleotide identities (99–99.5%) among them and 99–100% identity with ToBRFV isolates in different countries. A ToBRFV isolate (MZ130503) was selected for mechanical inoculation and to evaluate symptom severity responses of 13 commonly grown tomato cultivars in Saudi Arabia. All of the tomato cultivars showed a wide range of symptoms. The disease severity index of the tested cultivars ranged between 52% and 96%. The importance ToBRFV disease severity and its expanding host range due to its resistance breaking ability was discussed.

## 1. Introduction

Tomato (*Solanum lycopersicum* L.; family: Solanaceae) is one of the most popular and economically important vegetable crops worldwide [1]. In Saudi Arabia, tomato is considered one of the most economically important vegetable crops grown in different regions. The total area under tomato cultivation is estimated to be 12,454.3 hectares, producing 351,212.4 tons of fresh tomato fruits [2]. Tomato plants are affected by many phytopathogens including several phytopathogenic fungi, bacteria, viruses, nematodes, phytoplasma, parasitic higher plants, and viroids that severely affect the productivity of the crop [3,4].

Tomato plant is susceptible to many viral pathogens belonging to different genera, i.e., *Cucumovirus*, *Begomovirus*, *Potyvirus*, *Tospovirus*, *Polerovirus*, and *Tobamovirus* [5,6,7]. Tobamovirus is the largest genus of the family Virgaviridae, comprising 37 virus species including the most devastating plant viruses [8], the most famous Tobacco mosaic virus (TMV), the Tomato mosaic virus (ToMV), and the Cucumber green mottle mosaic virus (CGMMV) [9,10,11]. Tobamoviruses are spread through mechanical contact, such as workers’ hands, clothing, and farm tools, and they are transmissible through infected seeds and contaminated soil [9].

Recently, a new emerging viral pathogen that is spreading widely within greenhouses as well as in open fields of tomato crops, described as tomato brown rugose fruit virus (ToBRFV) [12], in the genus Tobamovirus, has been gaining more attention around the world. ToBRFV is an emerging threat, overcoming the genetic resistance that had been employed for more than 60 years against tobamoviruses in tomato. Since then, ToBRFV has spread worldwide, causing significant losses in tomato production [13]. All current findings indicate that this virus can be mechanically transmitted through infected sap using any method [6,14,15]. As a result, the virus has the potential to spread rapidly in greenhouses as well as in open fields.

The incidence and current distribution of ToBRFV infection affecting tomato have been reported from Jordan [12], then in Israel [16], Mexico [17], the United States [18], Germany [19], Italy [20], Palestine [21], Turkey [22], China [23], Egypt [24], Iran [25,26], Albania [27], Saudi Arabia [28], and Syria [29]. Additional reports also highlighted the presence of the virus in other European countries including the United Kingdom [30], the Netherlands [31], Greece [32], Spain [33], and France [34].

In the case of ToBRFV disease incidence in Saudi Arabia, after being reported from more than two dozen countries throughout the world, in January 2021, unusual fruit and leaf symptoms were observed in several greenhouses cultivating tomatoes commercially in Riyadh Region, Saudi Arabia, such as moderate-to-severe mosaic with dark green wrinkling, blistering, narrowing, and deformation with necrosis spot on tomato leaves, alongside irregular brown necrotic lesions, deformation, and yellowing spots on tomato fruits. The aim of this study was to identify the presence of tomato brown rugose fruit virus (ToBRFV) in Riyadh Region, Saudi Arabia, and evaluate the symptoms response of the most commercially grown tomato cultivars in Saudi Arabia in terms of mechanical inoculation by ToBRFV.

## 2. Results

### 2.1. Visual Inspection and Detection of Tomato Viruses Using ELISA

**Figure 1:**
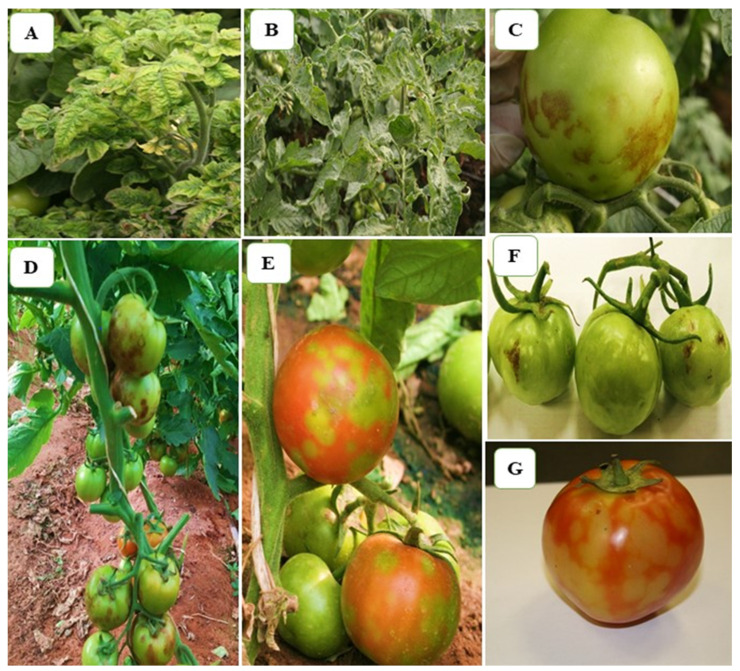
Symptoms of ToBRFV observed in different naturally infected tomato plants: (**A**,**B**) severe mosaic with dark green wrinkling, blistering, narrowing, and deformation on leaves; (**C**,**D**) irregular brown necrosis; (**E**,**G**) discoloration and yellow spots on fruits; (**F**) deformation on fruits.

The tomato plant symptoms induced by ToBRFV were wide and complex, wherein leaf symptoms showed moderate-to-severe mosaic with dark green wrinkling, blistering, narrowing, and deformation with necrosis spots on tomato leaves, while the fruit symptoms showed irregular brown necrotic lesions, deformation, and yellowing spots, rendering the fruits non-marketable, all observed in most of the tomato crops in many different locations in Riyadh Region (Figure 1).

All collected samples were tested with ELISA against ToCV, TSWV, TCSV, TAV, TBSV, TBRV, TRSV, ToMV, PepMV, TYLCV, and ToBRFV. The obtained results as indicated in Table 1 showed that 77.24% (112/145) of symptomatic tomato samples were found positive against at least one of the tested viruses by ELISA. The obtained results showed that 52.4% (76/145) of symptomatic tomato samples were found positive for ToBRFV, 12 out of 76 samples (6.9%) were infected with ToBRFV only, and 64 out of 145 (44%) had mixed infection between ToBRFV and at least one of the tested viruses.

### 2.2. Mechanical Inoculation and Host Range Determination

**Figure 2:**
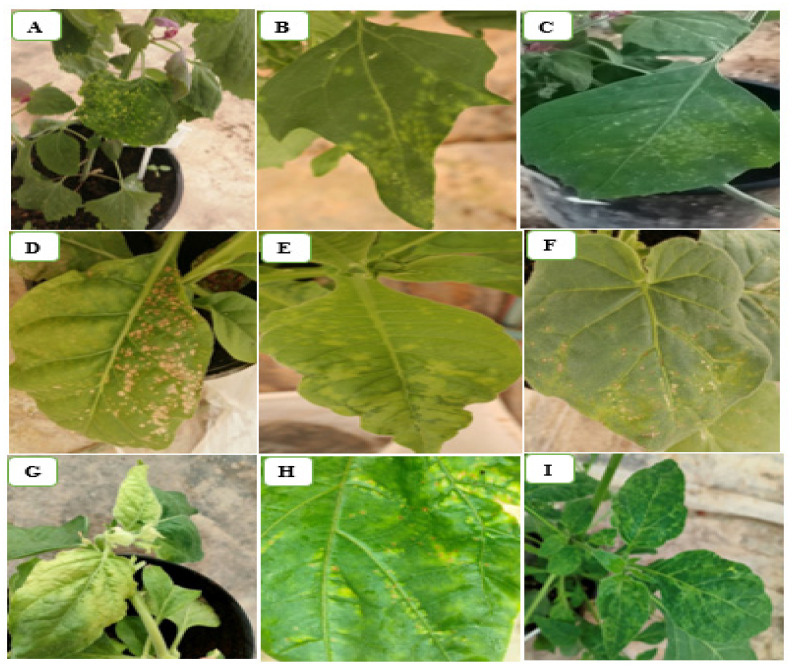
Reactions of selected host range plants showing different symptoms produced by ToBRFV inoculation: (**A**–**C**) chlorotic local lesions on the *C. amaranticolor*, *C. quinoa*, and *C. glucum* plants, respectively; (**D**,**E**) necrotic local lesions and severe mosaic on the *N. tabacum* plant, respectively; (**F**) necrotic local lesions on the *N. glutinosa* plant; (**G**) yellowing on the *N. benthamiana* plant; (**H**) necrotic ringspot on the *D. stramonium* plant; (**I**) mosaic on the *S. nigrum* plant.

The selected plant species for host range showed a wide range of symptoms compared with the non-inoculated control plants of the same species. Necrotic local lesions were developed on the inoculated leaves of *N. tabacum* and *N. glutinosa* plants. *N. benthamiana* and *N. occidentalis* showed yellowing, necrosis, and collapsed plants. On the other hand, *S. lycopersicum* plants showed leaf blistering, mosaic, narrowing, and leaf deformation, whereas *C. annum* plants showed symptoms of necrotic lesions in inoculated leaves and stems. Chlorotic local lesions were observed in the *Chenpodium* spp. (Figure 2). All these host plants showed positive reactions in DAS-ELISA and RT-PCR detection. *S. melongena* and *S. tuberosum* showed no symptoms and had a positive reaction when tested by double-antibody sandwich enzyme-linked immunosorbent assay (DAS-ELISA) and RT-PCR, while *C. lanatus*, *C. melo*, and *C. sativus* were asymptomatic but had a negative reaction when tested by the same techniques. All symptoms were recorded on inoculated (local symptoms) and non-inoculated leaves (systemic symptoms), as mentioned in Table 2.

### 2.3. Detection of ToBRFV by RT-PCR

**Figure 3:**
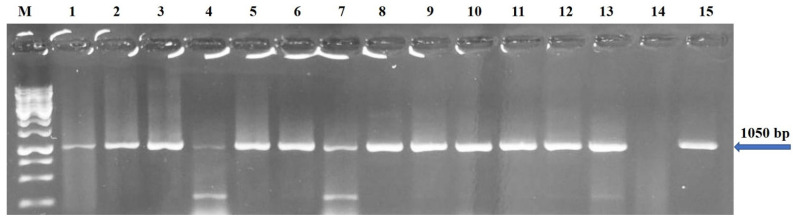
Analysis of a RT-PCR product (1052 bp) amplified using specific primers (F-3666 and R-4718) by RT-PCR from samples isolated from different locations (Al-Kharj (lanes 1 ), Az-Zulfi (lane 2), Al-Ghat (lane 3), Al-Quway’iyah (lane 4), Howtat Bani Tamim (lane 5), Al-Aflaj (lane 6), Al-Majmaah (lane 7), Ad-Dilam (lane 8), Al-Hareeq (lane 9), Huraymilla (lane 10), Al-Bark (lane 11), Al-Uyayna (lane 12) Ad-Dawadmi (lane 13), Lane 14 as anegstive control, Thadiq (lane 15), and Lane M, 1 Kb DNA Ladder (Thermo Fisher Scientific, Waltham, MA, USA).

The obtained results using PCR products from cDNA synthesized in the selected samples from different locations during the survey revealed that ToBRFV was detected when running on agarose gel electrophoresis (1%), giving specific bands (1052 bp), and it was clearly visible in all tested samples and their comparison with 1 Kb DNA Ladder (Thermo Fisher Scientific, Waltham, MA, USA), confirming the presence of ToBRFV in these samples (Figure 3).

### 2.4. Partial Genome Nucleotide Sequencing and Phylogenetic Analysis

**Figure 4:**
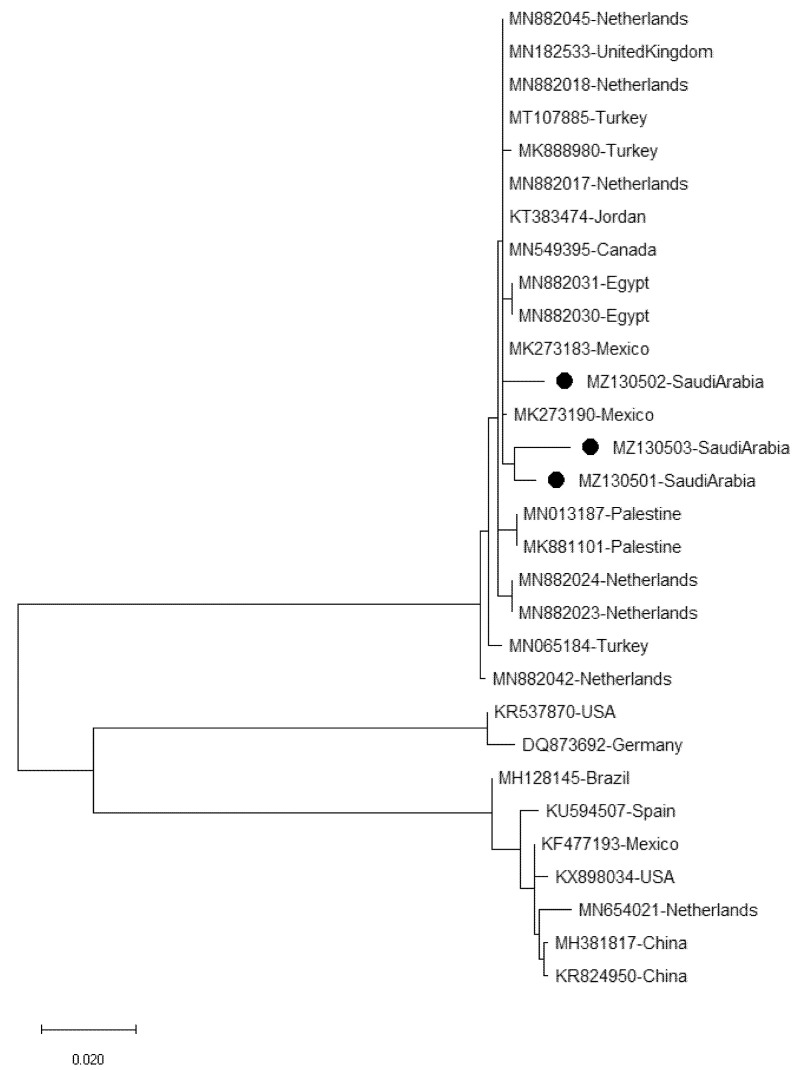
Phylogenetic tree based on multiple sequence alignment between three isolates of ToBRFV from Saudi Arabia and twenty-seven isolates documented in GenBank.

Sequences of the three selected isolates representing Riyadh Region (Al-Kharj, Az-Zulfi, and Al-Hareeq) were submitted to GenBank, named as ToBRFV-SA-F8, ToBRFV-SA-L10, and ToBRFV-SA-L24, respectively, and had the accession numbers MZ130501, MZ130502, and MZ130503, respectively. Sequences of three samples showed high similarity to all ToBRFV isolates that were registered in the NCBI. The data obtained from the phylogenetic tree revealed limited genetic variability among all Saudi isolates of ToBRFV and the sequences of other isolates available in the NCBI isolated from different host species and from different geographical countries. BLAST analysis of the three Saudi Arabian isolates of ToBRFV showed that the sequence shared nucleotide identities ranged between 99% and 99.5% among them and showed 98.9–99.9% identity with other GenBank isolates, including Palestine (MK881101 and MN013187), Turkey (MK888980, MN065184, and MT107885), the United Kingdom (MN182533), Egypt (MN882030 and MN882031), Jordan (KT383474), Mexico (MK273183 and MK273190), Canada (MN549395), and the Netherlands (MN882017, MN882018, MN882042, MN882023, MN882024, and MN882045). The lowest identity (82.1–82.8%) was found with the ToMMV isolates isolated from China (MH381817 and KR824950), the Netherlands (MN654021), Mexico (KF477193), the USA (KX898034), Spain (KU594507), and Brazil (MH128145), as well as 81.9–82.5% with ToMV isolates isolated from the USA (KR537870) and Germany (DQ873692) (Table 3). A homology tree was designed to compare the phylogenetic relationship between virus isolates (Figure 4).

### 2.5. Evaluation of Tomato Cultivars Responses to the Saudi Isolate of ToBRFV

**Figure 5:**
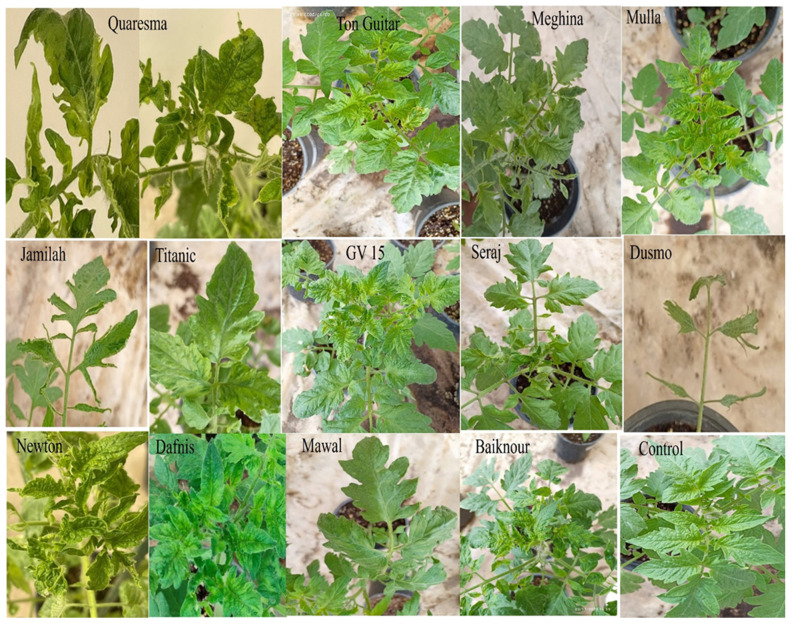
Symptoms on tomato cultivars inoculated by ToBRFV showing different symptoms on different cultivars.

**Figure 6:**
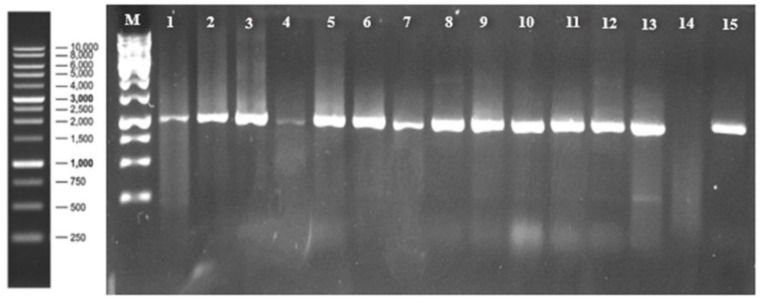
Gel electrophoreses (1%) showing positive RT-PCR amplification using F-3666- and R-4718-specific primers. (1052 bp) of ToBRFV from 13 tomato cultivars (lanes 1 to 13: tomato cultivars Dusmo, Seraj, Qurasema, Titanic, JV 15, Dafins, Baiknour, Jamilah, Tone Guitar, Newton, Mulla, Meghinha, Mawal, respectively); (M = 1 Kb DNA Ladder; Thermo Fisher Scientific, Waltham, MA, USA); (14 = negative control); (15 = positive control).

This experiment was carried out to evaluate the responses of thirteen commercial tomato cultivars to mechanical inoculation with a Saudi Arabian isolate of ToBRFV (accession no. MZ130503). Symptoms started to show up on upper and systemic new leaves in all cultivars after 2–3 weeks after inoculation. All of the tomato cultivars showed a wide range of symptoms including mosaic, mottling, leaf deformations, leaf narrowing, leaf rolling, blistering, and shoestring (Figure 5). The DSI ranged between 52% and 96% (Table 4). ToBRFV was detected in all thirteen tested commercial tomato cultivars by RT-PCR (Figure 6).

## 3. Discussion

ToBRFV is an emerging viral pathogen that is highly virulent, very aggressive, and fast spreading. ToBRFV belongs to the genus Tobamovirus and causes significant yield losses to tomato crop and its fruit quality as well, and therefore early detection is important in orderto thwart its spread [15]. In this study, various disease symptoms on tomato plants caused by ToBRFV in Riyadh Region, Saudi Arabia, were reported such as moderate-to-severe mosaic with dark green wrinkling, blistering, narrowing, and deformation with necrosis spots on tomato leaves, while irregular brown necrotic lesions, deformation, and yellowing spots rendering the fruits non-marketable were observed on tomato fruits. Symptoms of ToBRFV in tomato vary among environmental conditions and tomato cultivars [16]. In Jordan, ToBRFV causes brown rugose symptoms on tomato fruits, and hence the virus was named tomato brown rugose fruit virus [12]. Besides brown rugose, ToBRFV induced yellow spots on tomato fruits and brown rugose symptoms in some cultivars in Israel [16]. ToBRFV also caused yellow spots on fruits of tomato plants in Germany, Palestine, and China [19,21,35].

ELISA is considered as a valuable tool for virus detection and it is an easy test to deal with a large number of samples [36,37]. ELISA is a robust technique that enables the detection of viral capsid protein subunits of tobamoviruses. ELISA was adapted successfully for the detection of tobamoviruses, including ToBRFV. The ELISA test was conducted to screen the most common tomato viruses using kits that were available in our laboratory, including our survey, and the ELISA results clearly demonstrated that ToBRFV was present in commercial tomato crops collected from Riyadh Region, Saudi Arabia.

Various plants were proven to be experimental hosts to ToBRFV, and similar symptoms to those in our study were described by several investigators on *C. quinoa*, *C. amaranticolor*, *C. album*, *N. benthamiana*, *N. glutinosa*, *N. tabacum*, *S. lycopersicum*, *C. annum*, *S. nigrum*, and *D. stramonium* [16,21,35,38]. Petunia plants are symptomless hosts, while eggplants and potatoes are non-hosts for the virus [16]. On the other hand, ToBRFV causes latent infection on eggplants and potatoes [35]. Plants from the family Cucurbitaceae were found to be non-hosts for ToBRFV [35,38].

PCR and RT-PCR, which are specific and sensitive nucleic-acid-based methods for detection of plant viruses [39], were performed to diagnose viruses. All tissue samples (leaves or fruits) of the selected tomato plants tested positive for ToBRFV by RT-PCR, as the expected size of the 1052 bp fragment of the ORF encoding the RNA dependent RNA polymerase [16] was amplified, sequenced, and confirmed in terms of the presence of the virus in the study area. The main purpose of the molecular studies was to confirm the presence of ToBRFV in the study area and makeup of Saudi isolates of ToBRFV and their percentage identity and phylogenetic relationship with other isolates reported worldwide. The identity analyses and phylogenetic analysis of the Saudi isolates shared the highest nucleotide and amino acid identities with isolates from the Netherlands, Canada, the United Kingdom, and Jordan.

The Saudi isolate (MZ130503) infected all tested tomato cultivars and caused a wide range of symptoms including mosaic, mottling, leaf deformations, leaf narrowing, leaf rolling, blistering, and shoestring. DSI ranged between 52% and 96%. A similar study from China [35] reported that all the 50 tomato cultivars tested were highly sensitive to mechanical inoculation of ToBRFV, and systemic leaves of inoculated tomato plants showed symptoms of yellowing, curling, rolling, narrowing, blistering, and mosaic. In another study, all cultivated tomatoes and the great majority of wild tomatoes showed a wide range of symptoms including mosaic, leaf deformations, mottling, shoestring, and stunting in their reaction to ToBRFV inoculation [40].

## 4. Materials and Methods

### 4.1. Survey, Sample Collection, and Detection of Tomato Viruses Using ELISA

During the 2021–2022 growing season, a total of one hundred and forty-five symptomatic tomato leaves and fruits showed irregular brown necrosis, deformation, and yellowing spots on fruits samples, while mottling, mosaic with dark green wrinkling, and narrowing were collected from different locations in Riyadh Region (Al-Kharj, Az-Zulfi, AL-Ghat, Al-Quway’iyah, Howtat Bani Tamim, Al-Aflaj, Al-Majmaah, Ad-Dilam, Huraymilla, Al-Hareeq, Al-Bark, Ad-Dawadmi, Thadiq, and Al-Uyayna).

All collected samples were tested by double antibody sandwich enzyme-linked immunosorbent assay (ELISA) against the Tomato chlorosis virus (ToCV), Tomato spotted wilt virus (TSWV), Tomato chlorotic spot virus (TCSV), Tomato aspermy virus (TAV), Tomato bushy stunt virus (TBSV), Tomato black ring virus (TBRV), Tomato ringspot virus (TRSV), Tomato mosaic virus (ToMV), Pepino mosaic virus (PepMV), and Tomato brown rugose fruit virus (ToBRFV), while the Tomato yellow leaf curl virus (TYLCV) was tested by triple antibody sandwich (TAS) ELISA. The ELISA procedure was performed according to the manufacturer instructions. ELISA kits were obtained from LOEWE^®^ Biochemica, Sauerlach, Munich, Germany.

### 4.2. Mechanical Inoculation and Host Range Determination

For ToBRFV biological purification, a single local lesion technique was carried out by mechanical inoculation using *C. amaranticolor* as a local lesion host, whereas *N. tabacum* was used as a propagative host for the following experiments [41,42]. For mechanical inoculation, the plant leaves were dusted with carborundum. The extracted sap was passed through a cheesecloth and used to inoculate the selected 19 plant species belonging to different botanical families, namely, *S. lycopersicum*, *S. melongena*, *S. nigrum*, *S. tuberosum*, *C. annum*, *N. glutinosa*, *N. occidentalis*, *N. tabacum*, *N. benthamiana*, *C. amaranticolor*, *C. glaucum*, *C. quinoa*, *C. album*, *G. globosa*, *D. stramonium*, *P. hybrida*, *C. lanatus*, *C. melo*, and *C. sativus*. All inoculated plants were kept in an insect-proof cage inside a greenhouse for 2−3 weeks, and symptom expressions on each species were described and recorded. DAS-ELISA and RT-PCR were used to confirm the presence of the ToBRFV.

### 4.3. Total RNA Extraction and RT-PCR

Total RNA was extracted from selected samples collected from different locations in Riyadh Region (Al-Kharj, Az-Zulfi, AL-Ghat, Al-Quway’iyah, Howtat Bani Tamim, Al-Aflaj, Al-Majmaah, Ad-Dilam, Huraymilla, Al-Hareeq, Al-Bark, Ad-Dawadmi, Thadiq, and Al-Uyayna) on the basis of their positive reaction to DAS-ELISA for ToBRFV using the Thermo Scientific Gene JET Plant RNA Purification Mini Kit following the manufacturer’s instruction main protocol. Complementary DNA (cDNA) was synthesized with specific random primer R-4718 (5′-CAATCCTTGATGTG TTTAGCAC-3′) in 20 µL volume using a OneScript^®^ Plus cDNA Synthesis Kit. The conditions of amplification were 1 cycle at 50 °C for 15 min and 85 °C for 5 min. PCR was carried out using the specific primers F-3666 (5′ATGGTACGAACGGCGGCAG-3′) and R-4718 (5′-CAATCCTTGATGTG TTTAGCAC-3′), which amplified a fragment of 1052 bp of the open reading frame (ORF) encoding the RNA-dependent RNA polymerase (RdRp) [16]. The reaction mixture was performed in a 25 µL volume using a Thermo Scientific DreamTaq Green PCR Master Mix (2X). PCR was performed using the following parameters: 94 °C for 5 min as a heating step of Taq DNA polymerase, 94 °C for 30 s (denaturation), 56 °C for 30 s (annealing), and 72 °C for 1 min (extension) for 35 cycles, followed by final extension at 72 °C for 5 min. PCR products were analyzed by electrophoresis on a 1% agarose. A 1Kb DNA Ladder (Thermo Scientific, USA) was used to determine the size of DNA-amplified cDNA products.

### 4.4. Partial Genome Nucleotides Sequencing and Phylogenetic Analysis

RT-PCR products of the three selected tomato samples were sent to Macrogen Inc., Seoul, South Korea, for Sanger sequencing on strands through its entirety. The sequence results were analyzed through BLASTn for comparison with published ToBRFV gene RdRp sequences retrieved from the National Center for Biotechnology Information (NCBI). The construction of the phylogenetic tree from the aligned sequences was conducted by using MEGA X [43], applying the maximum likelihood method (ML) algorithm. ToBRFV-SA-F8, ToBRFV-SA-L10, and ToBRFV-SA-L24 isolates were compared with 27 different isolates obtained from GenBank from different hosts and countries, a homology tree was designed to compare the phylogenetic relationship among virus isolates, and pairwise nucleotide sequence identity tables were made.

### 4.5. Evaluation of Tomato Cultivar Responses to the Saudi Isolate of ToBRFV 

This experiment was carried out to evaluate the responses of thirteen commercially cultivated tomato cultivars (Newton, Quaresma, Dafnis, JV 15, Jamilah, Seraj, Mawal (Syngenta Basel Switzerland.), Baikonour, Mulla F1 (Westfrisian seeds Gorredijk, Netherlands), Dusmo F1, Meghina F1 (ISI SEMENTI Fidenza PR, Italy), Tone Gutar (Seminis vegetable seeds, Mumbai, India ), and Titanic (Huizer Zaden, Rilland, Netherlands) by mechanical inoculation using the Saudi isolate of ToBRFV (accession no. MZ130503). All inoculated plants were kept in an insect-proof cage inside a greenhouse as mentioned above. The severity of the symptoms was evaluated as per the following scale: mild (+), moderate (++), severe (+++), very severe (++++), and symptomless (−) [44]. The disease severity index (DSI) listed in Table 5 was calculated by the followed formula [40]:

DSI (%) = ∑_(e = 0) ^ 5 eRe × 100 ÷ 5N(1)

DSI = disease severity index; e = class; Re = number of plants in class (e); N = total number of plants. RT-PCR was used to confirm the presence of the ToBRFV using the newly developed systemic leaves [35,40].

## 5. Conclusions

In summary, on the basis of the results obtained from this study, ToBRFV induced moderate-to-severe mosaic with dark green wrinkling, blistering, narrowing, and deformation with necrosis spots on tomato leaves, while irregular brown necrotic lesions, deformation, and yellowing spots rendering the fruits non-marketable were observed on tomato fruits. These symptoms were similar to those described in other studies (Salem et al. (2016) [12] and Luria et al. (2017) [16]). In 145 symptomatic tomato samples collected from different locations in Riyadh Region, 52.4% were found to be positive for ToBRFV, 6.85% were singly infected, and 44.13% had mixed infection between ToBRFV and with at least one of tested viruses. In the host range experiment, the plants *S. lycopersicum*, *S. melongena*, *S. nigrum*, *S. tuberosum*, *C. annum*, *N. glutinosa*, *N. occidentalis*, *N. tabacum*, *N. benthamiana*, *C. amaranticolor*, *C. glaucum*, *C. quinoa*, *C. album*, *G. globose*, *D. stramonium*, and *P. hybrida* were found to be susceptible to ToBRFV, while, plants of *C. lanatus*, *C. melo*, and *C. sativus* of the family Cucurbitaceae were found to be non-susceptible to ToBRFV. All the thirteen tested commercially tomato cultivars grown in Saudi Arabia showed a wide range of symptoms, including mosaic, mottling, leaf deformations, leaf narrowing, leaf rolling, blistering, and shoestring in response to mechanical inoculation with a Saudi isolate of ToBRFV (accession no. MZ130503). On the basis of the obtained results in this present study, it was established that Tomato brown rugose fruit virus (ToBRFV) is associated with the commercial tomato crop from Riyadh Region, Saudi Arabia.

## Figures and Tables

**Table 1:** Detection of tomato viruses in tomato samples collected from different locations in Riyadh Region.

Locations	GPS Coordinates	Altitude MSL (M)	ELISA Results for Important Tomato Viruses													
			Total Samples	ToBRFV	PepMV	TBRV	ToMV	ToCV	TBSV	TRSV	TAV	TSWV	TCSV	TYLCV	Mixed Infection	ToBRFV Single Infection
Al-Kharj	24°13′58″ N	430	61	21	9	16	1	19	4	0	18	0	3	6	17	4
	47°18′0″ E															
Az-Zulfi	26°14′10″ N	660	29	13	6	4	6	2	2	1	1	5	1	1	12	1
	44°40′19″ E															
AL-Ghat	26°07′21″ N	630	14	14	6	6	7	3	0	1	1	0	0	0	9	5
	44°51′54″ E															
Al-Quway’iyah	24°05′13″ N	710	29	17	1	3	9	9	3	0	4	2	0	0	17	0
	45°29′23″ E															
Howtat Bani Tamim	23°17′0″ N	600	19	0	1	0	0	1	0	0	0	0	0	0	0	0
	46°47′55″ E															
Al-Aflaj	22°35′07″ N	560	1	2	2	0	0	0	0	0	0	0	0	0	2	0
	46°29′34″ E															
Al-Majmaah	25°47′29″ N	660	4	2	0	0	0	0	0	3	0	1	1	0	2	0
	44°57′56″ E															
Ad-Dilam	24°03′01″ N	460	3	4	0	2	0	1	0	0	0	1	0	0	2	2
	47°05′09″ E															
Huraymilla	25°03′34″ N	790	4	0	1	0	0	0	0	0	0	0	0	1	0	0
	46°06′52″ E															
Al-Hareeq	23°36′58″ N	680	1	1	0	1	0	1	0	0	0	1	0	0	1	0
	46°30′01″ E															
Al-Bark	23°17′29″ N	600	3	1	0	1	0	0	0	0	0	1	0	0	1	0
	46°49′55″ E															
Ad-Dawadmi	24°50′44″ N	860	1	1	0	1	0	0	0	0	0	0	1	0	1	0
	44°30′38″ E															
Thadiq	25°18′12″ N	710	2	0	0	0	0	0	0	0	0	0	0	0	0	0
	45°59′27″ E															
Al-Uyayna	24°54′5″ N	740	1	0	0	0	0	1	0	0	0	0	0	0	0	0
	46°21′11″ E															
Total			145	76	26	34	23	37	9	5	24	11	6	8	64	12
Percentage %			-	52.40%	17.90%	23.40%	15.90%	25.50%	6%	3.40%	17%	7.60%	4%	5.50%	44%	6.90%

**Table 2:** Local and systemic symptoms produced by ToBRFV inoculation on the tested host plants.

Plant Species and Common Name	Local Symptoms ^a^	Systemic Symptoms ^a^	ELISA ^b^	RT-PCR ^b^
*Chenopodium amaranticolor* (lamb’s quarters)	CLL	NS	+	+
*Chenopodium glaucum* (oak-leaved goosefoot)	CLL	NS	+	+
*Chenopodium album* (fat hen)	CLL	C, LD	+	+
*Chenopodium quinoa* (quinoa)	CLL	C, LD	+	+
*Nicotiana tabacum* (native tobacco)	NLL	SM	+	+
*Nicotiana glutinosa* (native tobacco)	N, NLL	PC	+	+
*Nicotiana benthamiana* (native tobacco)	N	Y, PC	+	+
*Nicotiana occidentalis* (native tobacco)	N	Y, N, PC	+	+
*Gomphrina globosa* (globe amaranth)	NRS	M	+	+
*Solanum nigrum* (black nightshade)	NRS	M	+	+
*Petunia hybrida* (petunia)	NS	M	+	+
*Datura stramonium* (jimsonweed)	NRS	NS	+	+
*Capsicum annum* (pepper)	NLL	NLL, M	+	+
*Solanum lycopersicum* (tomato)	NS	M, N, B, D	+	+
*Solanum tuberosum* (potato)	NS	NS	+	+
*Solanum melongena* (brinjal)	NS	NS	+	+
*Cucumis melo* (muskmelon)	NS	NS	−	−
*Citrullus lanatus* (watermelon)	NS	NS	−	−
*Cucumis sativus* (cucumber)	NS	NS	−	−

^a^ Symptoms observed on local and systemic leaves in host range plants. CLL = chlorotic local lesions; NS = no symptoms; C = chlorosis; LD = leaf deformed; NLL = necrotic local lesions; N = necrosis; Y = yellowing; PC = plant collapse; SM = serve mosaic; NRS = necrotic ringspot; M = mosaic; N = narrowing; B = blistering; D = deformation. ^b^ ELISA and RT-PCR for the presence of ToBRFV (+) = positive; (−) = negative.

**Table 3:** Similarity percentages of partial sequences of the RdRP gene of three Saudi isolates of ToBRFV with that of twenty-seven isolates of ToBRFV, ToMMV, and ToMV documented in GenBank.

Acc. No	Country	Isolate	Host	Saudi Arabia Isolates		
				MZ130501	MZ130502	MZ130503
				Sequence Similarity % Age		
MN882017	The Netherlands	38886230-B	*S. lycopersicum*	99.6	99.4	99.9
MN882018	The Netherlands	38886257-A	*S. lycopersicum*	99.6	99.4	99.9
MN549395	Canada	Ca1B	*S. lycopersicum*	99.6	99.4	99.9
MN882045	Netherlands	39563388-B	*S. lycopersicum*	99.6	99.4	99.9
MN182533	United Kingdom	TBRFV.21930919	*S. lycopersicum*	99.6	99.4	99.9
KT383474	Jordan	Tom1-Jo	*S. lycopersicum*	99.6	99.4	99.9
MT107885	Turkey	TBRFV-Ant-Tom	*S. lycopersicum*	99.6	99.4	99.9
MN882024	The Netherlands	39055711-A	*S. lycopersicum*	99.6	99.4	99.9
MK888980	Turkey	TOM07	*S. lycopersicum*	99.4	99.1	99.7
MK273190	Mexico	TAN6	*C. annuum*	99.5	99.3	99.8
MK273183	Mexico	TAN1	*S. lycopersicum*	99.6	99.4	99.9
MN882030	Egypt	39070022-A	*S. lycopersicum*	99.5	99.3	99.8
MN882031	Egypt	39070022-B	*S. lycopersicum*	99.5	99.3	99.8
MK881101	Palestine	Tom42-PAL	*S. lycopersicum*	99.1	98.9	99.4
MN013187	Palestine	F42-PAL	*S. lycopersicum*	99.1	98.9	99.4
MN882023	The Netherlands	38890029-B	*S. lycopersicum*	99.1	98.9	99.4
MN065184	Turkey	ToBRFVPep1	*C. annuum*	99.1	98.9	99.4
MN882042	The Netherlands	39563361-A	*S. lycopersicum*	99.1	98.9	99.4
MH381817	China	ToMMV- HN	*S. lycopersicum*	82.5	82.3	82.4
KR824950	China	ToMMV- YYMLJ	*C. annuum*	82.5	82.3	82.4
MN654021	The Netherlands	ToMMV- 19-02305	*C. annuum*	82.4	82.1	82.3
KF477193	Mexico	ToMMV- MX5	*S. lycopersicum*	82.6	82.4	82.5
KX898034	USA	ToMMV-CA16-01	*S. lycopersicum*	82.5	82.3	82.4
KU594507	Spain	ToMMV-VLC-1	*S. lycopersicum*	82.5	82.3	82.4
MH128145	Brazil	ToMMV-CpB1	*S. lycopersicum*	82.8	82.5	82.7
KR537870	USA	ToMV-99-1	*S. lycopersicum*	82.4	81.9	82
DQ873692	Germany	ToMV1-2	*S. lycopersicum*	82.5	82	82.1
MZ130501	Saudi Arabia	ToBRFV-SA-F8	*S. lycopersicum*	100	99	99.5
MZ130502	Saudi Arabia	ToBRFV-SA-L10	*S. lycopersicum*	-	100	99.3
MZ130503	Saudi Arabia	ToBRFV-SA-L24	*S. lycopersicum*	-	-	100

**Table 4:** Tomato plant symptoms observed on different tomato cultivars inoculated with the Saudi Arabian isolate of ToBRFV, as well as DSI, symptoms severity rating, and RT-PCR results after three weeks after inoculation.

Cultivar Name	Observed Symptoms ^a^	Symptoms Severity Rating ^b^	Disease Severity Index	RT-PCR ^c^
Dusmo	M, B, LR, D, S	++++	88%	+
Meghina	M, B, D	+++	72%	+
Newton	M, B, N, LR, D, S	+++	80%	+
Quaresma	M, B, N, LR, D, S	++++	96%	+
Dafnis-F1	M, B, N, D, S	++++	92%	+
JV 15	M, N, D	+++	80%	+
Baiknour	M, N, B, LR	+++	84%	+
Mulla	M, B, D, LR	++	64%	+
Tone guitar	M, LR, D	+++	80%	+
Mawal	Mo, LR	++	72%	+
Jamilah	Mo, N, LR, B, D, S	++++	88%	+
Seraj	M, B, N, D	++	72%	+
Titanic	Mo, N, D	+	52%	+

^a^ Symptoms observed on different tomato cultivars inoculated with the Saudi Arabian isolate of ToBRFV. M = mosaic; B = blistering; LR = leaf rolling; D = deformation; S = shoestring; N = narrowing; Mo = mottling. ^b^ + (mild), ++ (moderate), +++ (severe), and ++++ (very severe). ^c^ RT-PCR results for the presence of ToBRFV. (+) = positive.

**Table 5:** Scale of symptom severity index on the top leaves of the inoculated tomato plant.

Classes	Symptoms
0	No symptoms
1	Mild mosaic or mottling, followed by recovery
2	Mild mosaic or mottling with leaf deformation
3	Moderate mosaic or mottling and leaf deformed followed by leaf rolling
4	Severe mosaic or mottling, and leaf deformation
5	Severe mosaic or mottling, leaf deformation, and shoestring

## Data Availability

Partial genome sequences of ToBRFV under the accession numbers MZ130501, MZ130502, and MZ130503 are available in the NCBI Genbank database.

## References

[1] FAO (2020). World Food and Agriculture–Statistical Yearbook 2020.

[2] Statistics H. (2020). General Authority for Statistics: Kingdom of Saudi Arabi. https://www.stats.gov.sa/en/1060-0.

[3] Jones J., Zitter T., Momol T., Miller S. (2016). Compendium of Tomato Diseases and Pests.

[4] Agrios G.N. (2005). Chapter Eleven-Plant diseases caused by fungi. Plant Pathol..

[5] Scholthof K.B., Adkins S., Czosnek H., Palukaitis P., Jacquot E., Hohn T., Hohn B., Saunders K., Candresse T., Ahlquist P. (2011). Top 10 plant viruses in molecular plant pathology. Mol. Plant. Pathol..

[6] Oladokun J.O., Halabi M.H., Barua P., Nath P.D. (2019). Tomato brown rugose fruit disease: Current distribution, knowledge, and future prospects. Plant Pathol..

[7] Ong S.N., Taheri S., Othman R.Y., Teo C.H. (2020). Viral disease of tomato crops (*Solanum lycopesicum* L.): An overview. J. Plant Dis. Prot..

[8] ICTV (2019). International Committee on Taxonomy of Viruses (ICTV) Data Base Descriptions [WWW Document]. http://www.ictvonline.org.

[9] Broadbent L. (1976). Epidemiology and control of tomato mosaic virus. Annu. Rev. Phytopathol..

[10] Dombrovsky A., Tran-Nguyen L.T., Jones R.A. (2017). Cucumber green mottle mosaic virus: Rapidly increasing global distribution, etiology, epidemiology, and management. Annu. Rev. Phytopathol..

[11] Rybicki E.P. (2015). A Top Ten list for economically important plant viruses. Arch. Virol..

[12] LL N., Mansour A., Ciuffo M., Falk B., Turina M. (2016). A new tobamovirus infecting tomato crops in Jordan. Arch. Virol..

[13] Bernabé-Orts J.M., Torre C., Méndez-López E., Hernando Y., Aranda M.A. (2021). New Resources for the Specific and Sensitive Detection of the Emerging Tomato Brown Rugose Fruit Virus. Viruses.

[14] Dombrovsky A., Smith E. (2017). Seed Transmission of Tobamoviruses: Aspects of Global Disease Distribution. Advances in Seed Biology.

[15] Zhang S., Griffiths J.S., Marchand G., Bernards M.A., Wang A. (2022). Tomato brown rugose fruit virus: An emerging and rapidly spreading plant RNA virus that threatens tomato production worldwide. Mol. Plant Pathol..

[16] Luria N., Smith E., Reingold V., Bekelman I., Lapidot M., Levin I., Elad N., Tam Y., Sela N., Abu-Ras A. (2017). A new Israeli Tobamovirus isolate infects tomato plants harboring Tm-22 resistance genes. PLoS ONE.

[17] Camacho-Beltrán E., Pérez-Villarreal A., Leyva-López N., Rodríguez-Negrete E., Ceniceros-Ojeda E., Méndez-Lozano J. (2019). Occurrence of Tomato brown rugose fruit virus infecting tomato crops in Mexico. Plant Dis..

[18] Ling K.-S., Tian T., Gurung S., Salati R., Gilliard A. (2019). First report of Tomato brown rugose fruit virus infecting greenhouse tomato in the United States. Plant Dis..

[19] Menzel W., Knierim D., Winter S., Hamacher J., Heupel M. (2019). First report of Tomato brown rugose fruit virus infecting tomato in Germany. New Dis. Rep..

[20] Panno S., Caruso A., Davino S. (2019). First report of Tomato brown rugose fruit virus on tomato crops in Italy. Plant Dis..

[21] Alkowni R., Alabdallah O., Fadda Z. (2019). Molecular identification of Tomato brown rugose fruit virus in tomato in Palestine. J. Plant Pathol..

[22] Fidan H., Sarikaya P., Calis O. (2019). First report of Tomato brown rugose fruit virus on tomato in Turkey. New Dis. Rep..

[23] Yan Z.-Y., Ma H.-Y., Han S.-L., Geng C., Tian Y.-P., Li X.-D. (2019). First report of Tomato brown rugose fruit virus infecting tomato in China. Plant Dis..

[24] Amer M., Mahmoud S. (2020). First report of Tomato brown rugose fruit virus on tomato in Egypt. New Dis. Rep..

[25] Ghorbani A., Rostami M., Seif S., Izadpanah K. (2021). First report of Tomato brown rugose fruit virus in greenhouse tomato in Iran. New Dis. Rep..

[26] Esmaeilzadeh F., Koolivand D. (2022). Occurrence of tomato brown rugose fruit virus in tomato in Iran. J. Plant Pathol..

[27] Orfanidou C.G., Cara M., Merkuri J., Papadimitriou K., Katis N.I., Maliogka V.I. (2022). First report of tomato brown rugose fruit virus in tomato in Albania. J. Plant Pathol..

[28] Sabra A., Al-Saleh M.A., Al-Shahwan I.M., Amer M.A. (2021). First report of Tomato brown rugose fruit virus infecting tomato crop in Saudi Arabia. Plant Dis..

[29] Hasan Z., Salem N., Ismail I., Akel E., Ahmad A. (2022). First report of Tomato brown rugose fruit virus on greenhouse tomato in Syria. Plant Dis..

[30] Skelton A., Buxton-Kirk A., Ward R., Harju V., Frew L., Fowkes A., Long M., Negus A., Forde S., Adams I. (2019). First report of Tomato brown rugose fruit virus in tomato in the United Kingdom. New Dis. Rep..

[31] Van de Vossenberg B.T., Dawood T., Woźny M., Botermans M. (2021). First expansion of the public Tomato brown rugose fruit virus (ToBRFV) Nextstrain build; inclusion of new genomic and epidemiological data. PhytoFrontiers™.

[32] Beris D., Malandraki I., Kektsidou O., Theologidis I., Vassilakos N., Varveri C. (2020). First report of Tomato brown rugose fruit virus infecting tomato in Greece. Plant Dis..

[33] Alfaro-Fernández A., Castillo P., Sanahuja E., Rodríguez-Salido M., Font M.I. (2021). First report of Tomato brown rugose fruit virus in tomato in Spain. Plant Dis..

[34] Ministère de L’agriculture et de L’alimentation (2020). Virus ToBRFV: Le Ministère Con_Rme la Contamination de Tomates En Serre Dans le Finistère. https://agriculture.gouv.fr/virus-tobrfv-le-ministere-confirme-la-contamination-de-tomates-en-serre-dans-le-finistere.

[35] Yan Z.Y., Zhao M.S., Liu L.Z., Yang G.L., Chao G.E.N.G., Yanping T.I.A.N., LI X.D. (2021). Biological and molecular characterization of tomato brown rugose fruit virus and development of quadruplex RT-PCR detection. J. Integr. Agric..

[36] Ling K.S., Zhu H.Y., Jiang Z.Y., Gonsalves D. (2000). Effective application of DAS-ELISA for detection of grapevine leafroll associated closterovirus-3 using a polyclonal antiserum developed from recombinant coat protein. Eur. J. Plant Pathol..

[37] Lommel S.A., McCain A.H., Morris T.J. (1982). Evaluation of indirect enzyme-linked immunosorbent assay for the detection of plant viruses. Phytopathology.

[38] Chanda B., Gilliard A., Jaiswal N., Ling K.-S. (2021). Comparative analysis of host range, ability to infect tomato cultivars with Tm-22 gene, and real-time reverse transcription PCR detection of tomato brown rugose fruit virus. Plant Dis..

[39] Ichiki T.U., Shiba T., Matsukura K., Ueno T., Hirae M., Sasaya T. (2013). Detection and diagnosis of rice-infecting viruses. Front. Microbiol..

[40] Jewehan A., Salem N., Tóth Z., Salamon P., Szabó Z. (2021). Screening of *Solanum* (sections *Lycopersicon* and *Juglandifolia*) germplasm for reactions to the tomato brown rugose fruit virus (ToBRFV). J. Plant Dis. Prot..

[41] Kahn R.P., Monroe R.L. (1963). Detection of tobacco veinal necrosis strain of potato virus Y in *Solanum cardenasii* and *S. andigenum* introduced into United States. Phytopathology.

[42] Hill S.A., Hill S.A. (1984). Methods in Plant Virology. Methods in Plant Pathology.

[43] Kumar S., Stecher G., Li M., Knyaz C., Tamura K. (2018). MEGA X: Molecular evolutionary genetics analysis across computing platforms. Mol. Biol. Evol..

[44] Al-Shahwan I.M., Abdalla O.A., Al-Saleh M.A. (1995). Response of greenhouse-grown cucumber cultivars to an isolate of zucchini yellow mosaic virus (ZYMV). Plant Dis..

